# Generalized cerebral atrophy seen on MRI in a naturally exposed animal model for creutzfeldt-jakob disease

**DOI:** 10.1186/1479-5876-8-125

**Published:** 2010-11-26

**Authors:** Alexia L McKnight, Lawrence A Minkoff, Diane L Sutton, Bruce V Thomsen, Perry L Habecker, Raymond W Sweeney, Gary Smith, Constantin A Dasanu, Thomas E Ichim, Doru T Alexandrescu, Joel M Stutman

**Affiliations:** 1 Assistant Professor of Radiology, University of Pennsylvania School of Veterinary Medicine, New Bolton Center, Kennett Square, PA 19348, USA; 2 Executive Vice President, Fonar Corporation, Marcus Drive, Melville, NY, USA; 3 National Scrapie Program Coordinator, United States Department of Agriculture, Animal and Plant Health Inspection Service, Veterinary Services, Riverdale, MD 20737, USA; 4 United States Department of Agriculture, Animal and Plant Health Inspection Service, Veterinary Services Laboratories, Ames, IA 50010, USA; 5 Professor, The Pennsylvania Animal Diagnostic Laboratory System at New Bolton Center and the Laboratory of Pathology and Toxicology, School of Veterinary Medicine, University of Pennsylvania, 382 W. Street Road, Kennett Square, Pennsylvania 19348, USA; 6 Professor of Medicine & Chief, Section of Medicine, Department of Clinical Studies, New Bolton Center, 382 West Street Road, Kennett Square, PA 19348, USA; 7 Professor of Population Biology and Epidemiology & Chief, Section of Epidemiology and Public Health, Department of Clinical Studies, New Bolton Center, 382 West Street Road, Kennett Square, PA 19348, USA; 8 Saint Francis Hospital and Medical Center, Hartford, CT 06105, USA; 9 Medistem Inc., San Diego, CA 92101, USA; 10 Georgetown Dermatology, Washington, DC 20010, USA; 11 Professor and Chairman (Retired), Medical Computer Science Program, College of Health Related Professions, Downstate Medical Center, State University of New York, Brooklyn, NY, USA

## Abstract

**Background:**

Magnetic resonance imaging has been used in the diagnosis of human prion diseases such as sCJD and vCJD, but patients are scanned only when clinical signs appear, often at the late stage of disease. This study attempts to answer the questions "Could MRI detect prion diseases before clinical symptoms appear?, and if so, with what confidence?"

**Methods:**

Scrapie, the prion disease of sheep, was chosen for the study because sheep can fit into a human sized MRI scanner (and there were no large animal MRI scanners at the time of this study), and because the USDA had, at the time of the study, a sizeable sample of scrapie exposed sheep, which we were able to use for this purpose. 111 genetically susceptible sheep that were naturally exposed to scrapie were used in this study.

**Results:**

Our MRI findings revealed no clear, consistent hyperintense or hypointense signal changes in the brain on either clinically affected or asymptomatic positive animals on any sequence. However, in all 37 PrP^Sc ^positive sheep (28 asymptomatic and 9 symptomatic), there was a greater ventricle to cerebrum area ratio on MRI compared to 74 PrP^Sc ^negative sheep from the scrapie exposed flock and 6 control sheep from certified scrapie free flocks as defined by immunohistochemistry (IHC).

**Conclusions:**

Our findings indicate that MRI imaging can detect diffuse cerebral atrophy in asymptomatic and symptomatic sheep infected with scrapie. Nine of these 37 positive sheep, including 2 one-year old animals, were PrP^Sc ^positive only in lymph tissues but PrP^Sc ^negative in the brain. This suggests either 1) that the cerebral atrophy/neuronal loss is not directly related to the accumulation of PrP^Sc ^within the brain or 2) that the amount of PrP^Sc ^in the brain is below the detectable limits of the utilized immunohistochemistry assay. The significance of these findings remains to be confirmed in human subjects with CJD.

## Background

Scrapie was first reported in 1730 in sheep and goats and is the longest known transmissible spongiform encephalopathy (TSE) [1]. In the past two decades, TSEs have received much attention since ingestion of bovine spongiform encephalopathy (BSE) infected beef was causally linked to the variant form of CJD (vCJD) [2]. These TSE diseases are progressively debilitating and invariably fatal neurodegenerative diseases that have very long incubation periods and unique neuropathological changes. The most widely accepted cause of the TSE diseases is an abnormal prion protein, identified as PrP^Sc ^in the case of scrapie, which is a stereoisomer of the normal prion protein (PrP^C^).

Ante-mortem diagnosis of the TSE diseases, in general, has proven to be quite challenging. MRI has been useful in CJD patients -- with both the sporadic and variant forms. It is helpful in the exclusion of other neurodegenerative diseases as well as, in some cases, the positive diagnosis of sCJD or vCJD [3-6]. For example, in a study of 162 sCJD cases, bilateral basal ganglia hyperintensity was found to be 67% sensitive and 94% specific. MRI findings included hyperintense alterations and/or brain atrophy, alone or in combination with each other. A normal MRI without any hyperintense changes or atrophy was seen in 27.2% (44/162) patients[3]. A bilaterally symmetric hyperintense pulvinar, or the "hockey stick sign", is reported to be present in 80% of vCJD patients in some studies [4,5,7]. CJD patients, however, present with clinical symptoms at a relatively late stage of the disease.

MRI abnormalities are reported in pre-symptomatic mice experimentally infected (intraperitoneally) with scrapie. The study was performed at 9.4T, and a hyperintense septum and hippocampus were seen at 120 days post infection, approximately 60 days prior to the onset of clinical signs. Additional cortical and thalamic abnormalities were seen at 180 days post infection, when clinical signs became apparent [8].

Other MRI studies by Chung et al. in rodent scrapie models correlate MRI signal changes to neuoropathology [9,10]. One hamster model, performed at 4.7T, with scrapie induced intra-cerebral injections revealed a correlation with increased T2 signal and gliosis, and decreased T2 signal with vacuolization. In some areas with marked gliosis and vacuolization, no MRI signal changes were seen suggesting a T2 cancelling effect [10].

In contrast to Chung's findings in hamsters, Haik et al. found no association of MRI signal change in two CJD patients with gliosis and no clear association with spongiform change. There was, however, strong correlation of MRI signal change with accumulation of PrP^Sc ^in the both the sCJD and the vCJD patient [11].

Unlike experimentally induced scrapie rodent models that have a different course of disease than natural infection and CJD patients that present with an advanced stage of disease, a naturally exposed scrapie flock is typically composed of sheep in various stages of disease. For this reason, these animals are considered a good model to study MRI findings in scrapie as a model for the TSE diseases. Our objective was to study the consistent MRI findings in a large flock of scrapie positive animals as confirmed by immunohistochemistry. The purpose was twofold: 1) to better understand TSE diseases by evaluating the MRI finding in naturally infected sheep, and 2) to assess the accuracy of MRI in the detection of TSE in both symptomatic and asymptomatic sheep.

## Methods

### Flock information

One hundred eleven scrapie-exposed sheep with the scrapie susceptible QQ_171 _genotype were used in this study. The sheep originated from a single commercial scrapie flock in the Midwest United States that was to be depopulated for regulatory reasons. The flock was comprised of 62 black faced breeds (24 Hampshire, 39 Suffolk, 1 unknown black faced), 37 Western white faced sheep which entered the flock as adults prior to this study, and 12 brockel faced sheep born of the white faced ewes and black faced rams. The sheep ranged in age from 1-9 years old with the oldest sheep primarily the Western white faced breed.

Six additional sheep were purchased from two separate certified scrapie free flocks; five were black faced and one was white faced. They ranged in age from 2-8 years and served as known negative controls.

### MRI Examinations - Part I

Brain MR examinations were initially done on the 6 negative control animals and the 24 scrapie positive sheep which were identified by an immunohistochemistry test of surgically collected 3^rd ^eyelid lymph tissue from the 76 black and brockel faced sheep [12,13]. Each animal was scanned live under general anaesthesia and then recovered with the exception of the three most clinically affected animals, which were euthanized after the MRI exam, and one sheep that died during induction. We used a mobile 1T GE Signa LX MRI system (General Electric, Milwaukee, WI) with the general purpose flex coil wrapped around the dorsal and lateral aspects of the head. The following pulse sequences were obtained: T1- and T2-weighted fast spin echo, proton density (PD), inversion recovery (IR), fluid attenuated inversion recovery (FLAIR), and diffusion weighted imaging (DWI). The slice parameters were 3 mm thickness, 0 gap, 14 cm FOV for PD, T1, T2, IR, and FLAIR; for DWI 4 mm thickness, 0 gap, 22 cm FOV. Where possible 23 slices in the each of the axial, sagittal, and coronal planes were obtained.

### MRI Examinations - Part II

Based on the findings from Part I, all 113 remaining sheep were examined or re-examined by the same MRI protocol (with the exception of the T1 and FLAIR sequences) immediately following euthanasia.

### Quantitative Analysis

Lateral ventricle to cerebrum area ratios (V/C ratio) were calculated in all sheep. The V/C ratio is calculated by the following formula:

$$\begin{array}{l} \text{Lateral ventricle to cerebrum Ratio}=\text{Lateral ventricle area}/\text{(cerebrum area}-\\ \text{lateral ventricle area)}*\text{100 or, }\\ \text{more concisely: V}/\text{C}={\text{A}}_{V}{\text{/(A}}_{C}{\text{-A}}_{V}\text{)}*\text{100 and is reported as a percent}\text{.} \end{array}$$

**Figure 1:**
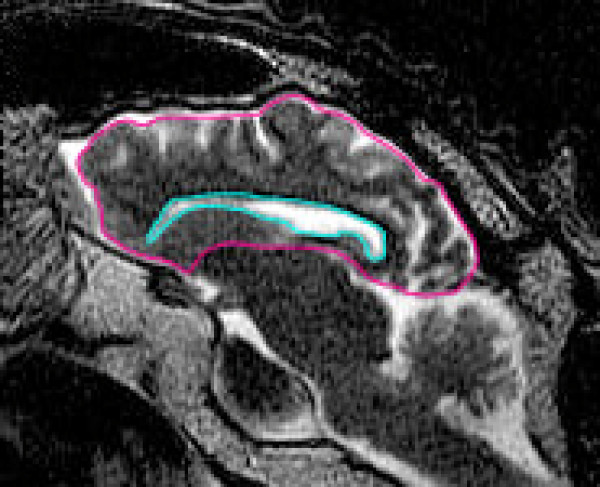
**The quantitative analysis in this study was performed by outlining the perimeter of the lateral ventricle and the cerebrum on a sagittal slice 3-5 mm from midline where the largest area of lateral ventricle was present**. The areas were determined and the ventricle to cerebrum ratio (V/C ratio) was then calculated.

It is the area of the lateral ventricle normalized to the area of the sheep's cerebrum area as imaged in that sagittal slice, and the value is reported as a per cent. It is an effort to measure the size of this sheep's lateral ventricle area as related to its own cerebrum area (without the ventricle area included in the cerebrum area). On the FSE T2 weighted sequences, a sagittal slice 3-5 mm off midline that had the largest lateral ventricular area was used for the measurements. As shown in Figure 1, regions of interest were drawn around the border of the lateral ventricle and, in the same slice, around the cerebrum.

Two scientists measured the areas using two different methods on different computers with no communication between them regarding their results. The scientists did their work in separate locations at different times with no communication regarding the V/C results. Both used mouse pointers to trace the outlines of the lateral ventricle and the cerebrum in the same slice as defined above.

Scientist A used Adobe's Photoshop software and their "Magnetic Lasso" technology with the following parameters: feather = zero pixels; anti-aliased = on; width = 3 pixels; edge contrast = 100%, and frequency = 100. Scientist B wrote his own software code in Microsoft Visual Basic 6.0 with DicomObjects.ocx as the DICOM interface and created a routine which counts pixels inside bounded planar regions. Dicomobjects.ocx is a library of compiled software enabling DICOM files to be studied using many different higher level programming languages for control. http://www.medicalconnections.co.uk.

Both techniques used exactly the same definition of the boundary between the cerebrum from the cerebellum, the only place in the slices of interest in which the boundary was less clear than all other tissue boundaries: this required a line drawing rule over that narrow region, which rule was used by both. Resulting percentages were similar, only varying by a small multiplicative constant; and, finally, both obtained similar graphs.

For the total data set of 117 sheep, the inter-observer reliability (correlation coefficient) between the scientist A and scientist B was 0.85 by the Pearson Product Moment Method and 0.87 by the Spearman Rank Order Method.

### Laboratory analysis

Scrapie testing by immunohistochemistry procedures followed the standard protocols used in the United States Department of Agriculture (USDA) scrapie eradication program and are similar to those described previously [13]. The pre-mortem third eyelid tissues were evaluated at the University of Wyoming (EW) and post-mortem sections of medulla at the obex, medial retropharyngeal lymph node and tonsil were examined at the National Veterinary Services Laboratory in Ames, IA (BT). Briefly, tissue sections were deparaffinized, rehydrated, treated with 95% formic acid (lymph tissue only) and then autoclaved in an antigen retrieval solution obtained from DakoCytomation, Carpinteria, CA, USA [14]. The sections were stained with an automated immunohistochemistry system (by Ventana Medical Systems, Tucson, AZ USA) which used a mixture of two monoclonal antibodies, F89/160.1.5 and F99/97.6.1, to detect prion protein [13]. Known positive and negative tissue samples were run as controls for each group of slides. The slides were interpreted independently of the MRI results. Later, additional areas of the brain (4 areas of the cerebrum and l section each of the thalamus, rostral colliculus, pons and cerebellum) were examined by IHC on eight of the nine animals which were found to be positive only on lymph tissue and negative on brain samples at the level of the obex.

All sheep were genotyped at codons 171, 136, and 154. There were only 4 genotypes present in the 117 sheep: 97 AARRQQ; 4 AARHQQ; 4 AARRQR; and 12 AVRRQQ.

### Clinical examinations

All 111 scrapie exposed sheep were evaluated clinically for neurological signs consistent with scrapie. The 24 eyelid positive animals were more thoroughly examined by recording the following parameters: body condition score, percent wool loss, presence of ataxia, and trembling.

### Statistical analysis

The extent to which the MRI results discriminate between "scrapie" and "not scrapie" was evaluated using a receiver operating characteristic (ROC) curve consisting of a graph of sensitivity versus one minus specificity as the cutoff is varied. The parameters and characteristics of the ROC curve was estimated from the data using STATA (Statacorp, 2001). The area under the ROC curve is used as a summary measure of the extent of the discrimination [15].

### Approvals

All aspects of this study were approved by the University of Pennsylvania's Institutional Animal Care and Use Committee, Environmental Health and Radiation Safety, and the Pennsylvania Department of Agriculture.

## Results

The clinical signs of scrapie, trembling and ataxia with various combinations of wool loss and/or thin body condition score, were only identified in 9 sheep. The other 102 sheep in the infected flock showed no detectable signs consistent with scrapie. IHC testing found 37 out of 111 sheep positive for scrapie with 9 of 37 sheep positive only on lymph tissues. Eight of these 9 lymph-only positive sheep remained PrP^Sc ^negative following additional IHC testing on multiple areas of the brain. Additional brain samples were unavailable for testing on the single remaining animal.

**Figure 2:**
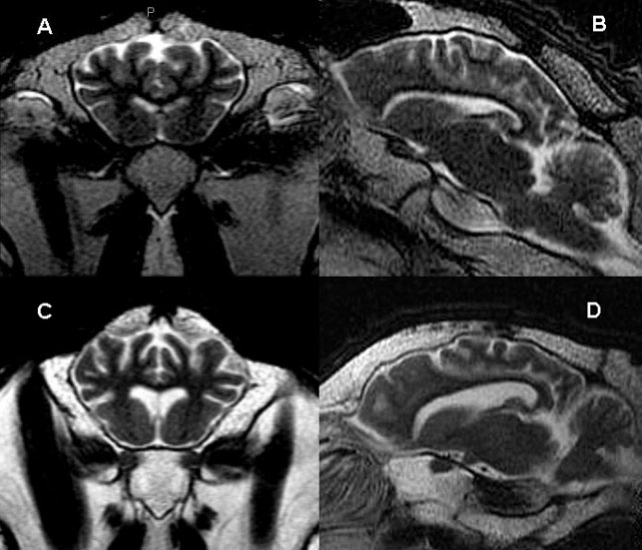
**Axial and sagittal MR images of a normal control sheep (A and B) compared to the most clinically affected animal in the study (C and D)**. The sulcal prominence and enlarged lateral ventricles indicative of diffuse cerebral atrophy are seen in the scrapie affected sheep.

The third eyelid test identified 24/76 (31%) sheep as PrP^Sc ^positive. Performing the eyelid test allowed an antemortem diagnosis to identify several scrapie infected sheep. In Part I of the study when brain MRI exams of these eyelid positive animals were compared to the 6 control animals, no clear, consistent MRI signal changes were noted in the brain of either the 9 clinically affected or the 15 asymptomatic sheep on any pulse sequence. As seen in Figure 2, the most severely affected clinical animals had hyperintense adipose tissue, predominantly within the medullary cavity of the skull and around the retropharyngeal lymph nodes, corresponding with serous atrophy secondary to emaciation. There was also mild subjective enlargement of the lateral ventricles with sulcal prominence in the most clinically affected sheep indicative of diffuse cerebral atrophy (Figure 2). This finding prompted quantitative evaluation in all sheep.

**Figure 3:**
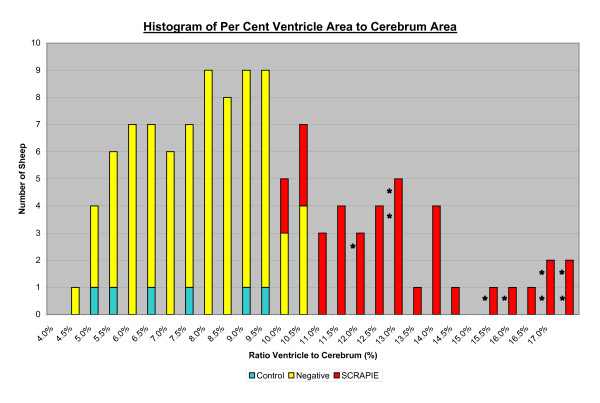
**The quantitative results of the study are displayed in this histogram**. The 37 PrP^Sc ^positive sheep (red) have larger V/C ratios relative to 74 PrP^Sc ^negative sheep (yellow) and 6 normal controls (blue) as defined by immunohistochemistry. Nine of the 37 positive sheep showed clinical signs of scrapie (asterisks).

**Figure 4:**
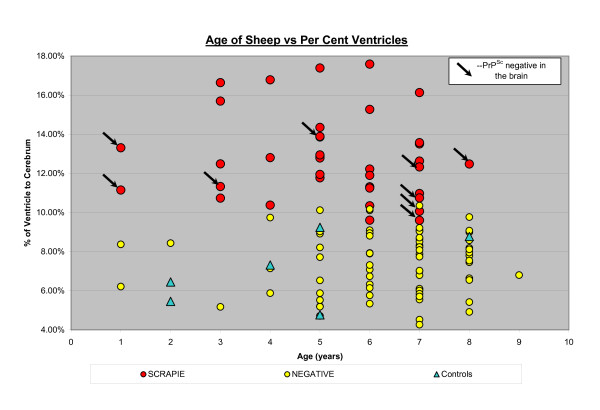
**No correlation of the lateral ventricle to cerebrum ratio with age was seen, only larger ratios in PrP^Sc ^positive sheep as displayed in red**. Nine of the 37 positive sheep, depicted by arrows, were PrP^Sc ^negative in the brain, but positive in the lymph tissue, including 2 one-year old animals.

The results of the quantitative analysis following Part II of the study are shown in Figures 3 and 4. The 37 PrP^Sc ^positive sheep had larger V/C ratios compared to the PrP^Sc ^negative sheep (Figure 3). Interestingly, 9 of these 37 sheep, including 2 one-year olds, were PrP^Sc ^positive in the retropharyngeal lymph nodes and/or tonsils but negative in the brain (Figure 4). As seen in Figure 4, no correlation with the V/C ratios with age was seen. Almost all animals with a V/C ratio over 15% showed clinical symptoms of scrapie.

The 37 PrP^Sc ^positive sheep fall into the following genotypes: 36 of 97 AARRQQ; 0 of 4 AARHQQ; 0 of 4 AARRQR; and 1 of 12 AVRRQQ. Every score above 10.4% corresponded to a PrP^Sc ^positive and every score below 9.5% corresponded to a negative: there was only a 10.25% overlap in scores, and most importantly only 8.75% false negatives when all scores are considered and the above cutoffs are not used.

In addition, it is noteworthy that the AVRRQQ subjects are, despite their relatively small N, the most ambiguous, in that 5 of the 11 negatives with this genotype fall into the upper quartile of all negative scores; this 'leaning' towards the high end of the negative distribution, might serve to suggest that over time this genotype might turn out to be the most likely to shift from negative to positive, and in future work should receive special attention regarding possible false negatives.

**Figure 5:**
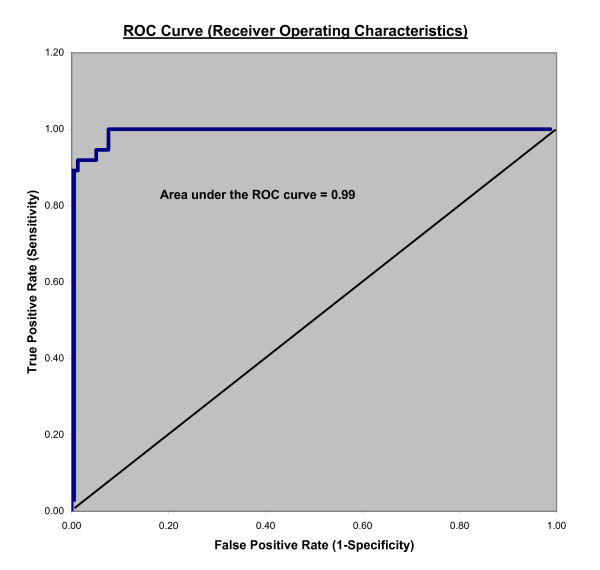
**ROC curve shows the V/C ratio is in the 'outstanding discrimination' range for correctly characterizing scrapie positive animals in this highly infected scrapie flock**.

The ROC curve is shown in Figure 5. The area under the curve was 0.99 (95% confidence interval, 0.98-1.00). As described in Hosmer and Lemeshow (page 162), this is in the "outstanding discrimination" range [15].

## Discussion

The 111 scrapie exposed QQ sheep used in this study are from a single commercial flock in the Midwest United States that had a high prevalence of infection (33% of the QQ animals were PrP^Sc ^positive on post-mortem IHC). Because there was a wide range in ages, multiple breeds, and clinical stages of the disease progression, this flock was considered a good model for evaluating MRI findings in scrapie positive sheep. The MRI findings correlated with IHC results in each of the breeds examined. The scrapie associated MRI changes detected subclinically infected animals and also detected 10 animals which were not identified by the current antemortem third eyelid test.

MRI signal abnormalities were not seen consistently on T2, FLAIR, or PD weighted images in this flock as reported in CJD patients and rodent scrapie models. Although similar inconsistencies are also seen in people, hyperintense changes in this study were a rare finding. Reasons for the hyperintense change in some animals and not others remain unclear. Meissner et al. found a correlation between the presence and absence of MRI findings and the CJD genotype in human patients [6]. The genotypes of this flock are very homogeneous and could explain the relative uniform lack of MRI signal abnormality.

The animals with the most severe clinical signs had the highest ventricular to cerebrum ratios; almost all animals with a V/C ratio over 15% showed clinical symptoms of scrapie. For this reason, we believe that enlarged ventricular to cerebral ratios may be positively correlated with disease progression. Similar correlations between brain atrophy as seen on MRI and progression of clinical disease have also been reported in other neurodegenerative diseases such as Alzheimer's disease [16] and multiple sclerosis [17,18].

Ventricular enlargement with sulcal prominence is typical of brain atrophy on MRI examinations [16,19,20]. This observation in the most clinically affected animals suggested similar evidence of cerebral atrophy/neuronal loss that has been reported in CJD patients[3] and a rodent scrapie model[10] with advanced disease. Particularly noteworthy was the finding, following quantitative analysis of all 117 sheep (111 scrapie exposed and 6 normal controls) that cerebral atrophy was a consistent finding in the 37 PrP^Sc ^animals, even among the asymptomatic sheep and, of particular interest, in the 2 positive one-year old sheep. The quantification of certain brain parameters on MR images, such as the V/C ratio as used in this study, may be considered as an ante-mortem tool for live animals at risk for scrapie, including young animals.

The pathophysiologic process that would explain diffuse cerebral atrophy in young asymptomatic sheep is unclear. The progression of scrapie in the naturally infected animal begins with an oral infection. Particularly susceptible in the perinatal period, lambs first show evidence of PrP^Sc ^in the Peyer's patches, medial retropharyngeal lymph nodes, mesenteric lymph nodes, and tonsils about 2-5 months after birth [21-23] In approximately 12-18 months, but as early as 9-10 months, PrP^Sc ^enters the central nervous system and can be first found in the obex of the medulla and the T8-T10 thoracic spinal cord segments [22,24] At the terminal stage of disease in clinically affected animals, usually between 2-5 years of age, neuropil vacuolation, astrocytosis, neuronal loss, and shrunken 'dark neurons' are seen in areas rostral to the medulla [25]. These histopathological findings are reported to be within the neocortex of the cerebrum (particularly centered around the superior frontal lobe gyrus) as well as the diencephalon and mesencephalon, cerebellum, and brain stem [25].

However, as shown in the pathogenesis studies, PrP^Sc ^within the cerebrum in asymptomatic sheep is not an expected finding [21,22,24,25]. Routine screening for scrapie on IHC is performed at the level of the obex of the medulla, because identification of PrP^Sc ^at this site has shown to be most sensitive for detection of scrapie in the earliest stages of CNS involvement [26,27]. Ersdal et al. found evidence of PrP^Sc ^in the cerebellum of 1/17 sheep and not in the obex [24]. Accumulation of PrP^Sc^, spongiform change and/or astrogliosis rostral to the obex in asymptomatic sheep was not reported and considered to be unusual [24,26-30]. Therefore, diffuse cerebral atrophy in the 28 asymptomatic sheep in this study is difficult to explain.

Furthermore, 9 of the 37 PrP^Sc ^positive sheep in this study that had higher V/C ratios compared to PrP^Sc ^negative sheep were only PrP^Sc ^positive in lymphoid tissue, not in the obex. Additional immunohistochemistry was negative on areas of the brain rostral to the obex in the 8 re-tested animals, which is consistent with the literature that the obex negative PrP^Sc ^sheep are in an early stage of disease that has not yet reached the central nervous system [22]. Two possible explanations are that the diffuse cerebral atrophy in this scrapie flock is either not directly related to the accumulation of PrP^Sc ^in the brain or that low levels of PrP^Sc ^in the obex are present but simply undetected by the IHC testing.

## Conclusions

We found a greater lateral ventricle to cerebrum area ratio in PrP^Sc ^positive sheep compared to PrP^Sc ^negative sheep in this large, naturally scrapie infected flock. There was no age correlation with the V/C ratios, only higher ratios in more clinically affected sheep in advanced disease. The results of this study indicate that there is diffuse cerebral atrophy/neuronal loss seen with MRI in naturally infected scrapie sheep in young animals and prior to the onset of clinical signs that appears related to the progression of the disease. These results also suggest that the cerebral atrophy/neuronal loss is not directly related to the accumulation of PrP^Sc ^within the brain, or that the amount of PrP^Sc ^in the brain is below the detectable limits of the immunohistochemistry assay. The significance of these findings remains to be confirmed in human subjects with CJD.

## Competing interests

The Authors declare that they have no competing interests.

## Authors' contributions

ALM--Primary Author, Study Staffing, Study Management. LAM--MRI data acquisition and analysis, JMS--MRI data acquisition and analysis, BVT--Immunohistochemistry analysis at NVSL, PLH--Pathological assistance at New Bolton Center, RWS--Clinical assessment and medical care of flock, GS--Statistical analysis, CAD--Clinical assessment and integration of the obtained data, TEI-- Integration of the data, DTA--Clinical integration of the data, DLS--Acquisition and coordination of infected flock. All authors have read and approved the final manuscript.

## References

[B1] Woolhouse ME Coen P Matthews L Foster JD Elsen JM Lewis RM Haydon DT Hunter N A centuries-long epidemic of scrapie in British sheep? Trends in Microbiology 2001 9 67 70 10.1016/S0966-842X(00)01912-0 11173245

[B2] Bruce ME Will RG Ironside JW McConnell I Drummond D Suttie A McCardle L Chree A Hope J Birkett C Transmissions to mice indicate that 'new variant' CJD is caused by the BSE agent Nature 1997 389 498 501 10.1038/39057 9333239

[B3] Schroter A Zerr I Henkel K Tschampa HJ Finkenstaedt M Poser S Magnetic resonance imaging in the clinical diagnosis of Creutzfeldt-Jakob disease Arch Neurol 2000 57 1751 1757 10.1001/archneur.57.12.1751 11115241

[B4] Collie DA The role of MRI in the diagnosis of sporadic and variant Creutzfeldt-Jakob disease Jbr-Btr 2001 84 143 146 11688725

[B5] Collie DA Summers DM Sellar RJ Ironside JW Cooper S Zeidler M Knight R Will RG Diagnosing variant Creutzfeldt-Jakob disease with the pulvinar sign: MR imaging findings in 86 neuropathologically confirmed cases Ajnr: American Journal of Neuroradiology 2003 24 1560 1569 13679271 PMC7973975

[B6] Meissner B Kortner K Bartl M Jastrow U Mollenhauer B Schroter A Finkenstaedt M Windl O Poser S Kretzschmar HA Zerr I Sporadic Creutzfeldt-Jakob disease: magnetic resonance imaging and clinical findings Neurology 2004 63 450 456 15314808 10.1212/01.wnl.0000136225.80445.c9

[B7] Collie DA Sellar RJ Zeidler M Colchester AC Knight R Will RG MRI of Creutzfeldt-Jakob disease: imaging features and recommended MRI protocol Clinical Radiology 2001 56 726 739 10.1053/crad.2001.0771 11585394

[B8] Collie DA The role of MRI in the diagnosis of sporadic and variant Creutzfeldt-Jakob disease Jbr-Btr: Organe de la Societe Royale Belge de Radiologie 2001 84 143 146 11688725

[B9] Sadowski M Tang CY Aguinaldo JG Carp R Meeker HC Wisniewski T In vivo micro magnetic resonance imaging signal changes in scrapie infected mice Neurosci Lett 2003 345 1 4 10.1016/S0304-3940(03)00319-7 12809974

[B10] Chung YL Williams A Beech JS Williams SC Bell JD Cox IJ Hope J MRI assessment of the blood-brain barrier in a hamster model of scrapie Neurodegeneration 1995 4 203 207 10.1006/neur.1995.0025 7583685

[B11] Chung YL Williams A Ritchie D Williams SC Changani KK Hope J Bell JD Conflicting MRI signals from gliosis and neuronal vacuolation in prion diseases Neuroreport 1999 10 3471 3477 10.1097/00001756-199911260-00002 10619628

[B12] Haik S Dormont D Faucheux BA Marsault C Hauw JJ Prion protein deposits match magnetic resonance imaging signal abnormalities in Creutzfeldt-Jakob disease 2002 797 799 12112094 10.1002/ana.10195

[B13] Bender S Alverson J Herrmann LM O'Rourke KI Histamine as an aid to biopsy of third eyelid lymphoid tissue in sheep Vet Rec 2004 154 662 663 10.1136/vr.154.21.662 15198315

[B14] O'Rourke KI Baszler TV Besser TE Miller JM Cutlip RC Wells GA Ryder SJ Parish SM Hamir AN Cockett NE Preclinical diagnosis of scrapie by immunohistochemistry of third eyelid lymphoid tissue Journal of Clinical Microbiology 2000 38 3254 3259 10970367 10.1128/jcm.38.9.3254-3259.2000 PMC87369

[B15] Hosmer DW Lemeshow S Applied Logistic Regression 2000 Second New York: John Wiley & Sons, Inc

[B16] Miller JM Jenny AL Taylor WD Race RE Ernst DR Katz JB Rubenstein R Detection of prion protein in formalin-fixed brain by hydrated autoclaving immunohistochemistry for the diagnosis of scrapie in sheep J Vet Diagn Invest 1994 6 366 368 7948209 10.1177/104063879400600315

[B17] Forstl H Zerfass R Geiger-Kabisch C Sattel H Besthorn C Hentschel F Brain atrophy in normal ageing and Alzheimer's disease. Volumetric discrimination and clinical correlations Br J Psychiatry 1995 167 739 746 10.1192/bjp.167.6.739 8829740

[B18] Liu C Edwards S Gong Q Roberts N Blumhardt LD Three dimensional MRI estimates of brain and spinal cord atrophy in multiple sclerosis 1999 10.1136/jnnp.66.3.323 PMC1736263 10084530

[B19] Losseff NA Wang L Lai HM Yoo DS Gawne-Cain ML McDonald WI Miller DH Thompson AJ Progressive cerebral atrophy in multiple sclerosis. A serial MRI study Brain 1996 119 Pt 6 2009 2019 10.1093/brain/119.6.2009 9010005

[B20] Yoshihara M Tsunoda A Sato K Kanayama S Calderon A Differential diagnosis of NPH and brain atrophy assessed by measurement of intracranial and ventricular CSF volume with 3 D FASE MRI Acta Neurochir Suppl 1998 71 371 374 9779233 10.1007/978-3-7091-6475-4_107

[B21] Thompson AJ Identification of brain atrophy with MRI in MS Mult Scler 1998 4 257 259 9762685 10.1177/135245859800400332

[B22] van Keulen LJ Vromans ME van Zijderveld FG Early and late pathogenesis of natural scrapie infection in sheep APMIS 2002 110 23 32 10.1034/j.1600-0463.2002.100104.x 12064252

[B23] van Keulen LJ Schreuder BE Vromans ME Langeveld JP Smits MA Pathogenesis of natural scrapie in sheep Archives of Virology - Supplementum 2000 57 71 11214935 10.1007/978-3-7091-6308-5_5

[B24] Andreoletti O Berthon P Marc D Sarradin P Grosclaude J van Keulen L Schelcher F Elsen JM Lantier F Early accumulation of PrP(Sc) in gutassociated lymphoid and nervous tissues of susceptible sheep from a Romanov flock with natural scrapie Journal of General Virology 2000 81 3115 3126 11086143 10.1099/0022-1317-81-12-3115

[B25] Ersdal C Ulvund MJ Benestad SL Tranulis MA Accumulation of pathogenic prion protein (PrPSc) in nervous and lymphoid tissues of sheep with subclinical scrapie Veterinary Pathology 2003 40 164 174 10.1354/vp.40-2-164 12637756

[B26] Wood JL McGill IS Done SH Bradley R Neuropathology of scrapie: a study of the distribution patterns of brain lesions in 222 cases of natural scrapie in sheep, 1982-1991 Vet Rec 1997 140 167 174 10.1136/vr.140.7.167 9055393

[B27] Wood JL McGill IS Done SH Bradley R Neuropathology of scrapie: a study of the distribution patterns of brain lesions in 222 cases of natural scrapie in sheep, 1982-1991 Veterinary Record 1997 140 167 174 10.1136/vr.140.7.167 9055393

[B28] Hamir AN Miller JM Schmerr MJ Stack MJ Chaplin MJ Cutlip RC Diagnosis of preclinical and subclinical scrapie in a naturally infected sheep flock utilizing currently available postmortem diagnostic techniques Journal of Veterinary Diagnostic Investigation 2001 13 152 154 11289211 10.1177/104063870101300209

[B29] Ryder SJ Spencer YI Bellerby PJ March SA Immunohistochemical detection of PrP in the medulla oblongata of sheep: the spectrum of staining in normal and scrapie-affected sheep Veterinary Record 2001 148 7 13 10.1136/vr.148.1.7 11200410

[B30] Kim H O'Rourke KI Walter M Purchase HG Enck J Shin TK Immunohistochemical detection of scrapie prion proteins in clinically normal sheep in Pennsylvania Journal of Veterinary Diagnostic Investigation 2001 13 89 91 11243373 10.1177/104063870101300120

